# Hospital Administration: Hospital Committees and Overwork

**Published:** 1908-02-22

**Authors:** A Matron


### HOSPITAL ADMINISTRATION: HOSPITAL COMMITTEES AND OVERWORK.

Sir,—In your issue of February 15 you publish an excellent article on "Overworked Matrons," every word of which is absolutely true; but you deal chiefly, I believe, with larger hospitals, whereas I think the smaller ones are more difficult to manage. I am matron of an infirmary containing 56 beds (the average number occupied being 52), an out-patient department, and a fair-sized Nurses' Home. I receive no help whatever, and I constantly experience the difficulties you speak of. To give sufficient supervision actual work must be neglected or some of it left until midnight, and if the work is done then inspection becomes lax. But—and herein lies the difficulty in a hospital of this size—though there is too much work for one, there is not enough for two. The sisters are unable to give any assistance, as they have quite enough to do in their wards. My committee are very good indeed; but I am sure they have very little idea of the work which has to be done day by day; in fact, I think very few outsiders understand what life in hospital means. Of course, there is your remedy—"resignation"; but in my case it is not feasible, and if I gave up the post there would be plenty of applicants only too willing to accept it. Therefore the only thing to be done is to struggle on and do one's best. Yours truly,

A Matron.

